# Sodium Bicarbonate as an Alternative to Heparin for Catheter Lock in Hemodialysis

**DOI:** 10.1016/j.ekir.2025.01.039

**Published:** 2025-02-01

**Authors:** Rafaela Sierth, Indianara Pires, Rafael Marques da Silva, Fabiana Baggio Nerbass

**Affiliations:** 1 Fundação Pró-Rim, Joinville, Santa Catarina, Brazil

To the Editor:

We read with great interest the editorial by van der Meulen, “Citrate, the Green Alternative to Heparin in Hemodialysis,”^1^ highlighting benefits of citrate. This study provides an important perspective on sustainable kidney care and the need for alternatives to heparin, which carries significant environmental and health-related burdens.

We would like to draw attention to another promising alternative, sodium bicarbonate, as a catheter-locking solution for dual-lumen catheters used in hemodialysis.

During the COVID-19 pandemic, heparin shortages and rising costs necessitated the exploration of alternatives. Inspired by the findings of El-Hennawy *et al.*,^2^ who demonstrated that sodium bicarbonate reduced catheter-related thrombosis and bloodstream infections compared with saline, we implemented its use in our hemodialysis unit in Southern Brazil. The antimicrobial and anticoagulant properties of sodium bicarbonate, combined with its low cost, made it appealing.

Based on these findings, we replaced heparin with 8.4% sodium bicarbonate as the catheter-lock solution in our hemodialysis unit in Southern Brazil. After 15 months of use without notable adverse effects, we conducted a retrospective analysis comparing the rates of catheter removal because of catheter-related thrombosis or bloodstream infections during these months with those during the previous 15 months using heparin. A total of 428 catheters were evaluated. We observed a significant reduction in catheter-related bloodstream infections rates (from 18.8% with heparin to 6.3% with sodium bicarbonate; *P* < 0.001), while maintaining comparable rates of catheter removal because of catheter-related thrombosis (27.6% vs. 29.6%; *P* = 0.09). In addition, this intervention resulted in a 92% cost reduction over 15 months, amounting to savings of US$3261.

Sodium bicarbonate has several advantages including the following: it is widely available, environmentally benign, and cost-effective, aligning with the goals of “green nephrology.” Its low carbon footprint indicates its potential as a sustainable alternative to heparin and citrate, particularly in resource-limited settings.

We acknowledge that our findings are based on a retrospective analysis and require further confirmation through well-designed prospective clinical trials. Nevertheless, we believe that our observations can serve as inspiration for future studies exploring sodium bicarbonate as a viable alternative to heparin and citrate in specific clinical scenarios.
